# Effect of Hyperthyroidism Control During Pregnancy on Maternal and Fetal Outcome: A Systematic Review and Meta-Analysis

**DOI:** 10.3389/fendo.2022.800257

**Published:** 2022-06-24

**Authors:** Jose Mario Alves Junior, Wanderley Marques Bernardo, Laura Sterian Ward, Danilo Villagelin

**Affiliations:** ^1^Postgraduate Course Internal Medicine, Campinas State University, Campinas, Brazil; ^2^School of Medicine, University of São Paulo, São Paulo, Brazil; ^3^Department of Evidence-Based Medicine, University of São Paulo, São Paulo, Brazil; ^4^Laboratory of Cancer Molecular Genetics, School of Medicine Sciences, Campinas State University, Campinas, Brazil; ^5^Endocrinology and Metabolism, Hospital of the Pontifical Catholic University of Campinas (PUC-Campinas), Campinas, Brazil

**Keywords:** hyperthyroidism, pregnancy, treatment, meta-analysis, maternal, fetal

## Abstract

**Context:**

Although the overt hyperthyroidism treatment during pregnancy is mandatory, unfortunately, few studies have evaluated the impact of treatment on reducing maternal and fetal outcomes.

**Objective:**

This study aimed to demonstrate whether treatment to control hyperthyroidism manifested during pregnancy can potentially reduce maternal-fetal effects compared with euthyroid pregnancies through a systematic review with meta-analysis.

**Data Source:**

MEDLINE (PubMed), Embase, Cochrane Library Central, LILACS/BIREME until May 2021.

**Study Selection:**

Studies that compared, during the gestational period, treated women with hyperthyroidism versus euthyroid women. The following outcomes of this comparison were: pre-eclampsia, abruptio placentae, fetal growth retardation, gestational diabetes, postpartum hemorrhage, low birth weight, stillbirth, spontaneous abortions, premature birth.

**Data Extraction:**

Two independent reviewers extracted data and performed quality assessments. Dichotomous data were analyzed by calculating risk differences (DR) with fixed and random effect models according to the level of heterogeneity.

**Data Synthesis:**

Seven cohort studies were included. The results of the meta-analysis indicated that there was a lower incidence of preeclampsia (p=0.01), low birth weight (p=0.03), spontaneous abortion (p<0.00001) and preterm birth (p=0.001) favouring the euthyroid pregnant group when compared to those who treated hyperthyroidism during pregnancy. However, no statistically significant differences were observed in the outcomes: abruptio placentae, fetal growth retardation, gestational diabetes mellitus, postpartum hemorrhage, and stillbirth.

**Conclusions:**

Our findings demonstrated that treating overt hyperthyroidism in pregnancy is mandatory and appears to reduce some potential maternal-fetal complications, despite there still being a residual risk of negative outcomes.

## Introduction

Hyperthyroidism, considering all causes, occurs in 0.2 to 1.3% of the population in countries where iodine intake is sufficient. Graves’ disease (GD) is the most frequent cause and has a high prevalence in women who often appear in reproductive age (1, 2).

Thyrotoxicosis can manifest in pregnancy in three forms: gestational thyrotoxicosis, subclinical hyperthyroidism, and overt hyperthyroidism. Gestational hyperthyroidism is a short form of thyrotoxicosis caused by hCG’s excessive stimulation of the thyroid gland. It is usually limited to the first trimester of pregnancy. It affects 1-3% of all pregnancies, especially in women with hyperemesis gravidarum and multiple gestations (3). Subclinical hyperthyroidism is defined by TSH under the standard limit with average T4/T3 values, respecting the TSH and T4/T3 pregnancy reference values by trimester (1, 3). Overt hyperthyroidism is rare, occurring in only 0.2% of pregnancies, and GD is its most common cause (3, 4).

Although there is consensus on the need for treatment of overt hyperthyroidism during pregnancy, only a few studies evaluated the impact of this treatment on maternal and fetal endpoints (3, 5–11). Most data are conflicting regarding pregnancy outcomes such as pre-eclampsia, growth restriction, low birth weight, miscarriage, and premature birth. Hence, we aimed to understand better the maternal-fetal effects of treating overt hyperthyroidism with anti-thyroid drugs (ATD) (methimazole and propylthiouracil) during pregnancy, reducing adverse outcomes. The well-known teratogenic effects of ATD are beyond the scope of this review.

## Methods

This study was conducted following the PRISMA statement (12). The research protocol was registered in the International Prospective Register of Systematic Reviews (http://www.crd.york.ac.uk/PROSPERO) under CRD42021242704.

### Database Search

The research was exclusively carried out using electronic databases [Medline (https://pubmed.ncbi.nlm.nih.gov/), EMBASE (https://www.embase.com/), Cochrane Library (https://www.cochranelibrary.com/), and Lilacs/Bireme (https://lilacs.bvsalud.org/)]. Searches for randomized controlled trials (RCTs), non-randomized trials (NRS), prospective or retrospective cohort studies were restricted to articles published in English or Portuguese without time limits. The research was developed following the PICO strategy, as follows: (P) women with hyperthyroidism during pregnancy; (I) antithyroid drugs; (C) euthyroid pregnancies and (O): pre-eclampsia, abruptio placentae, fetal growth retardation, gestational diabetes, postpartum hemorrhage, low birth weight, stillbirth, spontaneous abortions, premature birth. Only publications with complete data were included, and the last search was conducted in May 2021. Literature searches were performed in PubMed as follows: (Pregnant Women OR Pregnant Woman OR Pregnancy OR Pregnacies OR Gestation OR Gravidity) AND (Hyperthyroidism OR Hyperthyroid OR Hyperthyroids OR Primary Hyperthyroidism OR Thyrotoxicosis OR Thyrotoxicosis OR Graves disease OR Basedow Disease OR Graves’ Disease OR Exophthalmic Goiter OR Exophthalmic Goiters OR Hyperthyroidism, Autoimmune OR Basedow’s Disease OR Basedows Disease) AND (Abortion OR Spontaneous Abortions OR Early Pregnancy Loss OR Early Pregnancy Losses OR Miscarriage OR Miscarriages OR Tubal Abortions OR Pre-Eclampsia OR Pre Eclampsia OR Preeclampsia OR Pregnancy Toxemias OR Pregnancy Toxemia OR Edema-Proteinuria-Hypertension Gestosis OR Edema Proteinuria Hypertension Gestosis OR Hypertension-Edema-Proteinuria Gestosis OR Hypertension Edema Proteinuria Gestosis OR Toxemia Of Pregnancy OR Toxemia Of Pregnancies OR EPH Complex OR EPH Toxemias OR EPH Toxemia OR Preeclampsia Eclampsia 1 OR Preeclampsia Eclampsia 1s OR Proteinuria-Edema-Hypertension Gestosis OR Proteinuria Edema Hypertension Gestosis OR Premature Birth OR Premature Births OR Preterm Birth OR Preterm Births OR Premature Infant OR Preterm Infants OR Preterm Infant OR Premature Infants OR Neonatal Prematurity OR Premature Obstetric Labor OR Premature Labor OR Preterm Labor OR Premature Obstetric Labor OR Abruptio Placentae OR Placental Abruption OR Placental Abruptions OR Fetal Growth Retardation OR Intrauterine Growth Retardation OR Intrauterine Growth Restriction OR Fetal Growth Restriction OR Diabetes, Gestational OR Pregnancy-Induced Diabetes OR Gestational Diabetes OR Gestational Diabetes Mellitus OR Postpartum Hemorrhage OR Immediate Postpartum Hemorrhage OR Delayed Postpartum Hemorrhage OR Stillbirth OR Stillbirths OR Low-Birth-Weight Infant OR Low Birth Weight Infant OR Low-Birth-Weight Infants OR Low Birth Weight OR Low Birth Weights). The same medical subject headings (MeSH) and keywords in various combinations were used in the mentioned electronic databases.

### Study Selection

Two reviewers performed independent eligibility assessments to select the studies using predefined inclusion and exclusion criteria. Any divergence was resolved by consensus or consulting a third reviewer. The inclusion criteria were (I) pregnant women who have been diagnosed and treated with hyperthyroidism during pregnancy and for whom at least one pregnancy outcome has been assessed and (II) randomized controlled trials (RCTs) or non-randomized trials (NRS) or prospective or retrospective cohort studies with ATD treatment in one comparison arm regardless of the patients’ number. The exclusion criteria were: (I) non-human studies, (II) letters, reviews, case reports, editorials, (III) studies without full text, and (IV) studies from which the necessary data could not be extracted from the pooled results.

### Quality Assessment

Study quality was assessed using the Newcastle-Ottawa scale to assess the quality of non-randomized studies in meta-analyses, and certainty assessment was performed using GRADE (13, 14). Disagreements were discussed between the investigators until a consensus was reached.

### Data Extraction

One reviewer extracted all relevant information from acceptable studies, including design, sample size, population details, recruitment process, hyperthyroidism exposure, method of treatment, and outcomes. If data were reported in separate metrics, extracted outcome data were converted to a standard metric to estimate treatment effects.

### Statistical Analysis

Statistical analyses were performed using the Review Manager software, version 5.4 (RevMan 5.4; Cochrane Collaboration, Oxford, UK). Dichotomous data were analyzed by computing risk differences (RD) with fixed- and random-effect models employed according to the level of heterogeneity. Sensitivity analysis with funnel plot for ≥50% heterogeneity was not performed because, as a rule of thumb, tests for funnel plot asymmetry should be used only when at least ten studies are included in the meta-analysis. Also, the power of the tests is low when there are fewer studies.

## Results

### Study Selection

**Figure 1:**
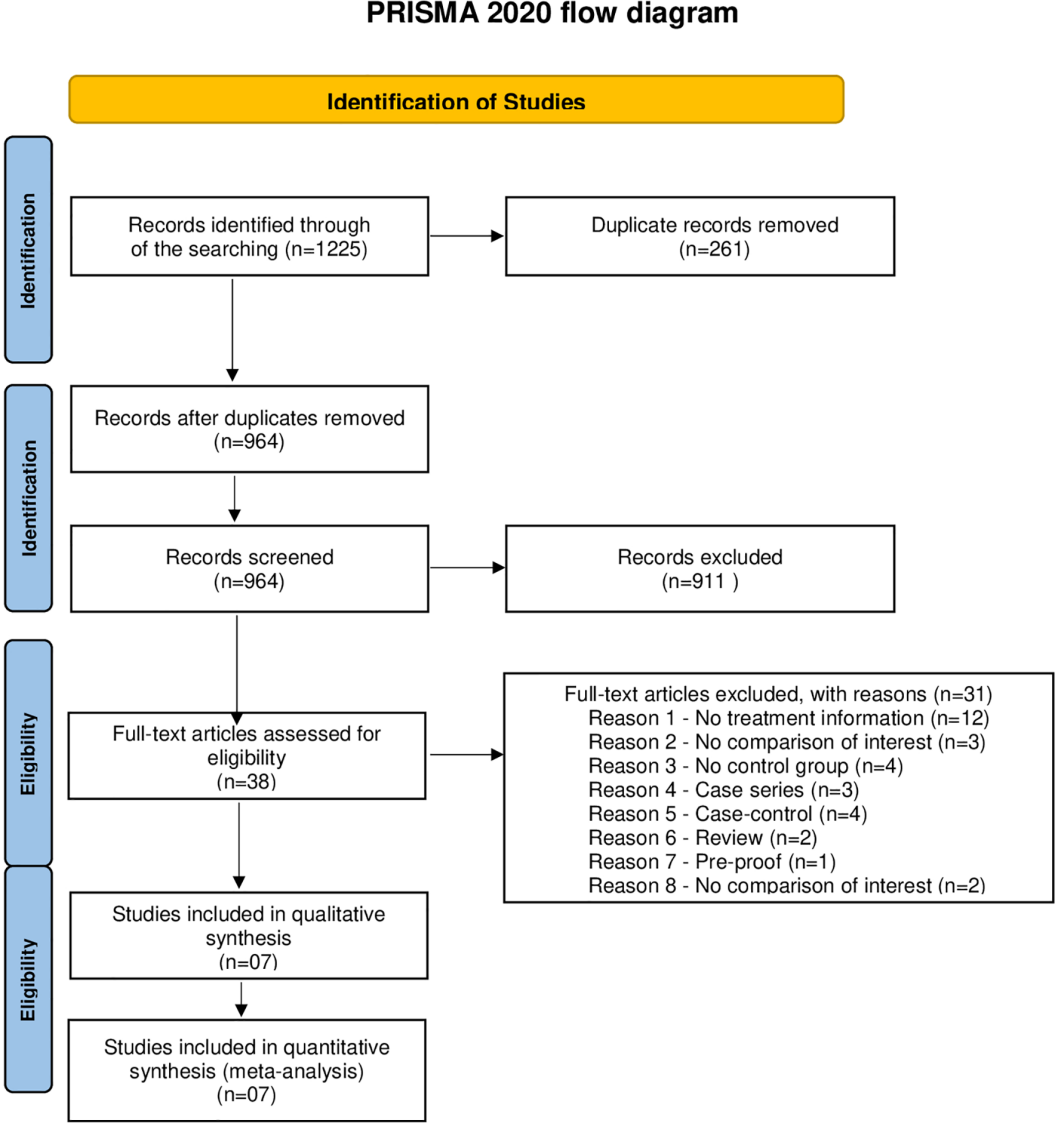
Prisma Flow Diagram of Study Selection.

After searching five databases and exploring reference lists, 1,225 potential studies were identified. The studies were uploaded to Endnote, where duplicates were excluded. After the exclusions, seven studies contained enough data to be included in a meta-analysis (**Figure 1**).

### Quality Assessment

All seven studies were considered as high quality by the Newcastle-Ottawa scale, as they scored between 7 and 8 (**Table 1**). Due to the high score, all studies were included in the systematic review and meta-analysis.

**Table 1:** Newcastle-Ottawa quality assessment scale.

Author	Year	Country	Design	Founding Source	Participants (Database)	Mean(age)	Study Size (n)	Intervention Groups	N	Control Groups	N	Folow-up (years)	Hypertireoidism During Pregnancy (Definition)
Ajmani SN et al. (5)	2014	India	Prospective Cohort	Not declared	Department of obstetrics and gynecology at Kasturba Hospital	27,53	400	Hyperthyroidism treated	2	Euthyroidism	347	01	High Free T4 ([2.0 ng/dl) with decreased TSH (\0.2 lIU/l)
Bánhindy F et al. (6)	2011	Hungary	Retrospective Cohort	Both nonindustrial and industrial	Hungarian Congenital Abnormality Registry (HCAR)	25.5	60,994	Hyperthyroidism treated	187	Euthyroidism	60.807	16	Thyrotoxicosis with diffuse goitre [Graves’ disease, toxic diffuse goitre], Thyrotoxicosis with toxic single thyroid nodule, and Thyrotoxicosis with toxic multinodular goitre
Luewan S et al. (7)	2011	Thailand	Retrospective Cohort	None	Maternal– Fetal Medicine Unit, Chiang Mai University and medical records of the patients.	28.98	563	Hyperthyroidism treated	180	Euthyroidism	360	14	Pregnant women diagnosed for hyperthyroidism by endocrinologist based on the clinical manifestations and endocrine laboratory confirmation
Pillar N et al. (8)	2010	Israel	Retrospective Cohort	None	Soroka University Medical Cente	26.5	185,825	Hyperthyroidism treated	189	Euthyroidism	185.636	19	Overactive thyroid gland, resulting in the overproduction and, thus, excess of circulating free thyroid hormones (triiodothyronine, thyroxine, or both)
Sahu MT et al. (9)	2010	India	Prospective Cohort	None	King George Medical University, and all India Institute of Medical Sciences	26.0	633	Hyperthyroidism treated	5	Euthyroidism	552	03	Elevation in free T4 with an undetectable serum TSH
Turunen S et al. (10)	2020	Finland	Retrospective Cohort	None	Medical Birth Register (MBR) and supplemented with information from the Prescription Register, the Hospital Discharge Register and the Register on Congenital Malformations.	27,5	571,785	Hyperthyroidism treated	580	Euthyroidism	550.860	09	The ICD-10 code E05 (all digits) and the ICD-9/ICD-8 code both 242
Andersen SL et al. (11)	2014	Denmark	Retrospective Cohort	None	Danish nationwide registers	30.0	1,062,862	Hyperthyroidism treated	5.229	Euthyroidism	836.905	11	ICD-8: 242.00–242.29 and ICD-10: E05.0–E05.9 [excluding thyrotoxicosis factitia (E05.4), overproduction of TSH (E05.8A) and thyrotoxic heart disease (E05.9A)].

### Studies Characteristics

All included studies were based on a retrospective cohort conducted in India, Hungary, Thailand, Israel, Finland, and Denmark. The sample sizes ranged from 400 to 1,062,862, and the mean age of studies ranged from 25.5 to 30. The definition of hyperthyroidism during pregnancy varied among the included studies, but the studies clearly expressed the treatment of these women with antithyroid drugs (**Table 2**).

**Table 2:** Baseline studies characteristics.

Cohortstudies	Selection				Comparability	Outcome			TOTAL (0 – 9)	Quality
	Representativeness of the exposed cohort	Selection of the nonexposed cohort	Ascertainment of exposure	Outcome of interest not present at start of study	Control for importantfactor or additional factor	Assessment of outcome	Follow-up for enough form outcome to occour	Adequacy of follow-up of cohorts		
Ajmani SN et al., 2014 (5)	*	*	*	*	*	*	*	*	8	High
Bánhindy F et al., 2011 (6)	*	*	*		*	*	*	*	7	High
Luewan S et al., 2011 (7)	*	*	*		*	*	*	*	7	High
Pillar N et al., 2010 (8)	*	*	*		*	*	*	*	7	High
Sahu MT et al., 2010 (9)	*	*	*	*	*	*	*	*	8	High
Turunen S et al., 2020 (10)	*	*	*		*	*	*	*	7	High
Andersen SL et al., 2014 (11)	*	*	*		*	*	*	*	7	High

The symbol "*" in each spot means that the characteristic's answer was present in the evaluated article.

### Study Findings

No study included the assessment of all eligible outcomes. Data describing the presence of pre-eclampsia cases were available in six out of the seven eligible trials studies; data on gestational diabetes mellitus in five studies; fetal growth retardation, stillbirth, and premature birth in four studies; abruptio placentae data were available in three studies and spontaneous abortion, postpartum hemorrhage, and low birth weight in only two studies.

### Meta-Analysis of Selected Studies

**Figure 2:**
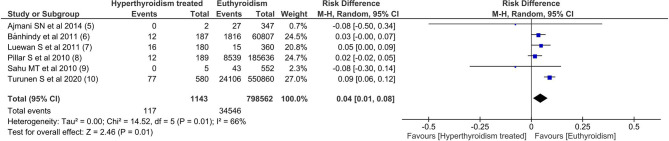
Forest Plot: Pre-eclampsia.

**Figure 3:**
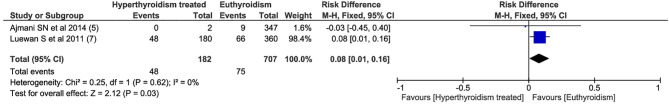
Forest Plot: Low Birth Weight.

**Figure 4:**
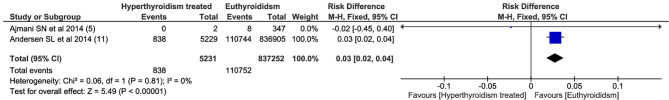
Forest Plot: Spontaneous Abortions.

**Figure 5:**
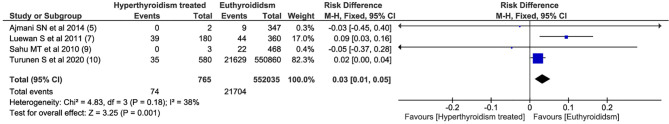
Forest Plot: Premature Birth.

Among all outcomes evaluated, only preeclampsia, low birth weight, miscarriage and preterm delivery showed a statistically significant difference. The pooled data from the network meta-analysis showed that euthyroid pregnant women, compared to pregnant women who underwent treatment for hyperthyroidism during pregnancy, had a lower incidence of preeclampsia: 4.3% vs. 10.2% (RD=0.04; 95% CI: 0.01 to 0.08; I^2^ = 66%; p=0.01) (**Figure 2**); low birth weight fetuses: 10.6% vs. 26.4% (RD=0.08; 95% CI: 0.01 to 0.16; I^2 ^= 0%; p=0.03) (**Figure 3**); spontaneous abortions: 13.6% vs. 16% (RD=0.03; 95% CI: 0.02 to 0.04; I^2 ^= 0%; p< 0.00001) (**Figure 4**) and, premature birth: 4.0% vs. 9.8% (RD=0.03; 95% CI: 0.01 to 0.05; I^2 ^= 38%; p=0.001) (**Figure 5**). Random-effects analysis method was used to adjust for inter-study heterogeneity and certainty assessment was very low for all outcomes (**Table 3**)

**Table 3:** GRADE - certainty assessment.

** Certainty assessment**							**№ of patients**		**Effect**		**Certainty**
**№ of studies**	**Study design**	**Risk of bias**	**Inconsistency**	**Indirectness**	**Imprecision**	**Other considerations**	**Anti-thyroid drugs (ATD)**	**No treatment**	**Relative**	**Absolute**	
									**(95% CI)**	**(95% CI)**	
**Pre-eclampsia**											
6	observational studies	not serious	not serious	not serious	serious^a,b^	none	117/1143 (10.2%)	34546/798562 (4.3%)	not estimable	**40 fewer per 1.000**	⨁◯◯◯
										(from 80 fewer to 10 fewer)	Very low
**Low Birth Weight**											
2	observational studies	not serious	not serious	not serious	serious^a^	none	48/182 (26.4%)	75/707 (10.6%)	not estimable	**80 fewer per 1.000**	⨁◯◯◯
										(from 160 fewer to 10 fewer)	Very low
**Spontaneous Abortions**											
2	observational studies	not serious	not serious	not serious	serious^a^	none	886/5231 (16.9%)	110928/837252 (13.2%)	not estimable	**30 fewer per 1.000**	⨁◯◯◯
										(from 40 fewer to 20 fewer)	Very low
**Premature Birth**											
4	observational studies	not serious	not serious	not serious	serious^a^	none	74/765 (9.7%)	21704/552035 (3.9%)	not estimable	**30 fewer per 1.000**	⨁◯◯◯
										(from 50 fewer to 10 fewer)	Very low
**Abruptio Placentae**											
3	observational studies	not serious	not serious	not serious	serious^a^	none	15/771 (1.9%)	14373/736843 (2.0%)	not estimable	**0 fewer per 1.000**	⨁◯◯◯
										(from 10 fewer to 10 more)	Very low
**Fetal Growth Retardation**											
4	observational studies	not serious	not serious	not serious	serious^a,b^	none	30/376 (8.0%)	4951/186895 (2.6%)	not estimable	**20 fewer per 1.000**	⨁◯◯◯
										(from 90 fewer to 50 more)	Very low
**Gestational Diabetes**											
5	observational studies	not serious	not serious	not serious	serious^a^	none	78/963 (8.1%)	59618/798202 (7.5%)	not estimable	**20 fewer per 1.000**	⨁◯◯◯
										(from 30 fewer to 0 fewer)	Very low
**Postpartum Hemorrhage**											
2	observational studies	not serious	not serious	not serious	serious^a^	none	4/182 (2.2%)	28/707 (4.0%)	not estimable	**0 fewer per 1.000**	⨁◯◯◯
										(from 30 fewer to 30 more)	Very low
**Stillbirth**											
4	observational studies	not serious	not serious	not serious	serious^a^	none	19/5991 (0.3%)	4453/1388472 (0.3%)	not estimable	**0 fewer per 1.000**	⨁◯◯◯
(from 0 fewer to 0 fewer)										Very low

“a” means “Type of study design” and “b” means “Heterogeneity > 50%”.

For the other outcomes evaluated, the pooled data of the network meta-analysis did not show statistically significant difference between the groups, as follows: abruptio placentae (RD=0.00; 95% CI: -0.01 to 0.01; I^2 ^= 0%; p=0.94), fetal growth retardation (RD=0.02; 95% CI: -0.05 to 0.09; I^2 ^= 67%; p=0.61), gestational diabetes mellitus (RD=0.02; 95% CI: -0.00 to 0.03; I^2 ^= 13%; p=0.08), postpartum hemorrhage (RD=-0.00; 95% CI: -0.03 to 0.03; I^2 ^= 0%; p=0.95) and stillbirth (RD=-0.00; 95% CI: -0.00 to 0.00; I^2 ^= 0%; p=0.34). Random-effects analysis method was used to adjust for inter-study heterogeneity when necessary and certainty assessment was also very low for all outcomes (**Table 3**).

## Discussion

According to the 2017 ATA guidelines, poor control of thyrotoxicosis is associated with pregnancy loss, pregnancy-induced hypertension, prematurity, low birth weight, intrauterine growth restriction, stillbirth, thyroid storm, and maternal congestive heart failure (3). Unfortunately, there are little data on the effect of controlling thyrotoxicosis during pregnancy on maternal outcomes. Moreover, the risk for adverse maternal outcomes in women who had overt hyperthyroidism treated with ATD during pregnancy differs in various studies, which may be due to differences in inclusion criteria, sample size, and study design (5–11).

The present meta-analysis compared almost 6,000 pregnant women treated for hyperthyroidism with 1.3 million euthyroid pregnant women, demonstrating that the treatment for hyperthyroidism and restoration of the euthyroid state can supposedly reduce the incidence of five essential outcomes: placental abruption, delayed fetal growth, gestational diabetes, postpartum hemorrhage, stillbirth.

Pre-eclampsia is a pregnancy complication characterized by high blood pressure and signs of damage to another organ system, most often the liver and kidneys. Pre-eclampsia incidence range from 2 - 7,5%; some risk factors are hypertension, obesity, diabetes mellitus, age, and race (15, 16). Hyperthyroidism is a well-known risk factor for pre-eclampsia, especially poorly controlled (5, 17). In addition, hyperthyroidism could aggravate a preexisting condition (e.g., hypertension) by predisposing to pre-eclampsia, or it can even trigger pre-eclampsia. Our data show that the development of preeclampsia was 4% lower in the pregnancies of euthyroid women.

Gestational diabetes mellitus is also an important outcome with several impacts on maternal health. Obesity is the major risk factor for diabetes mellitus (15, 16, 18). Also, hyperthyroidism is a well-known cause of increased insulin resistance and glucose levels. Both hormonal and immunologic conditions are related to this phenomenon (19). This meta-analysis suggests that overt hyperthyroidism treatment mitigates the deleterious effect of excessive thyroid hormone on glucose metabolism, preventing an increased risk of gestational diabetes mellitus (5, 6, 8–10).

Spontaneous abortion is a tragic situation during pregnancy. Chromosomal abnormality is the single most common cause involved in approximately half of all cases of early spontaneous abortion and is also related to stillbirth (20). In addition, uncontrolled hyperthyroidism during pregnancy is a risk factor, although the molecular mechanism underlying this association is still not well understood (3). Although small but significant, our results showed that euthyroid pregnant women had 3% fewer events of spontaneous abortions.

One of the critical consequences of uncontrolled hyperthyroidism during pregnancy is low birth weight, which may be directly or indirectly related to maternal hyperthyroidism (21). Maternal hyperthyroidism may reduce fetal nutrition or act as a predisposing factor for other conditions that will cause these outcomes, similar to pregnancy-induced hypertension and maternal congestive heart failure (22). In women with GD, maternal TRAb passage to the placenta can induce low birth weight (3). Although overt hyperthyroidism was medicated with ATD, and presumably it was controlled; euthyroid pregnant women had 8% lower occurrence of low birth weight.

Placental abruption is the early separation of the placenta from the lining of the uterus before completing the second stage of labor (23). The primary cause is impairment of the vascular structures that support the placenta (24). Hyperthyroidism can predispose to hypertension, one of the most critical risk factors of placental abruption (3). Correcting hyperthyroidism during pregnancy attenuates this predisposition, resulting in no differences between hyperthyroid-treated women and euthyroid controls (5, 8, 10).

Postpartum hemorrhage is the most common cause of maternal mortality worldwide, and the hyperthyroid state contributes to coagulation disorders, acting directly on the gene transcription of coagulation proteins and altering the clot’s structure (25, 26). In addition, hyperthyroidism may cause a hyper-dynamic state and favor bleeding. Our data demonstrated that controlling hyperthyroidism equals the risk of postpartum hemorrhage compared to the control group (5, 7).

The World Health Organization defines premature birth as births before 37 completed weeks of gestation (27). Premature birth rates range from 3 to 14% in low-risk pregnancies. Maternal conditions, including hyperthyroidism, and other conditions such as pre-eclampsia, pre-gestational and gestational diabetes, and cervical incompetence may increase this incidence (28, 29). This meta-analysis showed that even with the treatment of hyperthyroidism, the occurrence of preterm birth was 3% lower in euthyroid pregnant women (5, 7, 9, 10).

This meta-analysis shows an excess risk of hyperthyroid-treated women in four endpoints (pre-eclampsia, low birth weight, spontaneous abortion, and premature birth) in comparison to euthyroid women. The differences were slight, ranging from 3 to 8%, however significant. The deleterious effect of hyperthyroidism affects the three trimesters of gestation, in the first-trimester spontaneous abortion and during the second and third-trimester pre-eclampsia, low birth weight, and premature birth. The mechanisms involved may be several and may be directly or indirectly related to hyperthyroidism, as discussed above. Also, other causes can be related to these negative outcomes, such as other autoimmune conditions associated with GD (30). Also, GD TRAb could have essential participation in negative outcomes. TRAb passage through the placenta and the action in the thyroid fetus` gland also can determine the increase of these outcomes (31–33), especially in the third trimester. None of the studies evaluated in the meta-analysis mentioned the TRAb titers during treatment. The control of hyperthyroidism and the decrease in TRAb titers do not always present in the same period (34); thus, TRAb can cause fetus’ hyperthyroidism even with maternal thyroid levels normal.

Despite our attempts to eliminate potential biases, this systematic review has limitations. First, because some studies did not specify if hyperthyroidism was treated, we had to exclude many patients from the final analyses (35–46). Second, it is important to emphasize that in carrying out this meta-analysis, we only used studies that showed the treatment of overt hyperthyroidism during pregnancy. Unfortunately, some studies are not specific about the degree of control of hyperthyroidism, we presumed that an euthyroid state was reached and maintained in all treated patients. Also, some studies may have started treatment after the first trimester. Third, the quality of evidence evaluated by the GRADE tool showed a very low certainty of the evidence for all outcomes. The main weak point in the quality of evidence was the type of study design, leading to a high level of imprecision. In addition, we were unable to examine data on subclinical hyperthyroidism and gestational thyrotoxicosis, limiting this meta-analysis to overt hypothyroidism.

No meta-analysis or systematic review has been published on this topic to our knowledge. In conclusion, treatment of overt hyperthyroidism in pregnancy is mandatory and appears to reduce some potential maternal-fetal complications. However, there is a residual risk of negative results even when overt hyperthyroidism is treated. This information will help doctors and patients manage pregnancy, especially those who needed to treat hyperthyroidism during this process.

## Data Availability Statement

The original contributions presented in the study are included in the article/supplementary material. Further inquiries can be directed to the corresponding author.

## Author Contributions

JA: made considerable contributions to the design and postulation of the study, the definition of technical content, literature research, data analysis, statistical analysis, manuscript preparation, drafting, writing, critical review, and approval of the manuscript final version for publication. WB: were involved in the data analysis, statistical analysis, manuscript preparation, writing, drafting, critical review for important intellectual content. LW: Manuscript preparation, writing, drafting, critical review for important intellectual content, and approval of the manuscript final version for publication. DV: provided support for the entire process of developing and reviewing this systematic review and approval of the manuscript final version for publication. All authors contributed to the article and approved the submitted version.

## Conflict of Interest

The reviewer FEP declared a shared affiliation with the author DV to the handling editor at the time of review.

The remaining authors declare that the research was conducted in the absence of any commercial or financial relationships that could be construed as a potential conflict of interest.

## Publisher’s Note

All claims expressed in this article are solely those of the authors and do not necessarily represent those of their affiliated organizations, or those of the publisher, the editors and the reviewers. Any product that may be evaluated in this article, or claim that may be made by its manufacturer, is not guaranteed or endorsed by the publisher.

## References

[1] Taylor PN Albrecht D Scholz A Gutierrez-Buey G Lazarus JH Dayan CM . Global Epidemiology of Hyperthyroidism and Hypothyroidism. Nat Rev Endocrinol (2018) 14(5):301–16. doi: 10.1038/nrendo.2018.18 29569622

[2] Hollowell JG Staehling NW Flanders WD Hannon WH Gunter EW Spencer CA . T(4), and Thyroid Antibodies in the United States Population (1988 to 1994): National Health and Nutrition Examination Survey (NHANES III). J Clin Endocrinol Metab (2002) 87(2):489–99. doi: 10.1210/jcem.87.2.8182 11836274

[3] Alexander EK Pearce EN Brent GA Brown RS Chen H Dosiou C . Guidelines of the American Thyroid Association for the Diagnosis and Management of Thyroid Disease During Pregnancy and the Postpartum. Thyroid (2017) 27(3):315–89. doi: 10.1089/thy.2016.0457 28056690

[4] Mestman JH . Evaluating and Managing Postpartum Thyroid Dysfunction. Medscape Women's Health (1997) 2(7):3. 9746699

[5] Ajmani SN Aggarwal D Bhatia P Sharma M Sarabhai V Paul M . Prevalence of Overt and Subclinical Thyroid Dysfunction Among Pregnant Women and its Effect on Maternal and Fetal Outcome. J Obstet Gynaecol India (2014) 64(2):105–10. doi: 10.1007/s13224-013-0487-y PMC3984645 24757337

[6] Bánhidy F Puhó EH Czeizel AE . Possible Association Between Hyperthyroidism in Pregnant Women and Obstructive Congenital Abnormalities of Urinary Tract in Their Offspring–A Population-Based Case-Control Study. J Matern Fetal Neonatal Med (2011) 24(2):305–12. doi: 10.3109/14767058.2010.487142 20504076

[7] Luewan S Chakkabut P Tongsong T . Outcomes of Pregnancy Complicated With Hyperthyroidism: A Cohort Study. Arch Gynecol Obstet (2011) 283(2):243–7. doi: 10.1007/s00404-010-1362-z 20087627

[8] Pillar N Levy A Holcberg G Sheiner E . Pregnancy and Perinatal Outcome in Women With Hyperthyroidism. Int J Gynaecol Obstet (2010) 108(1):61–4. doi: 10.1016/j.ijgo.2009.08.006 19766207

[9] Sahu MT Das V Mittal S Agarwal A Sahu M . Overt and Subclinical Thyroid Dysfunction Among Indian Pregnant Women and Its Effect on Maternal and Fetal Outcome. Arch Gynecol Obstet (2010) 281(2):215–20. doi: 10.1007/s00404-009-1105-1 19437026

[10] Turunen S Vääräsmäki M Lahesmaa-Korpinen AM Leinonen MK Gissler M Männistö T . Maternal Hyperthyroidism and Pregnancy Outcomes: A Population-Based Cohort Study. Clin Endocrinol (Oxf) (2020) 93(6):721–8. doi: 10.1111/cen.14282 32657434

[11] Andersen SL Olsen J Wu CS Laurberg P . Spontaneous Abortion, Stillbirth and Hyperthyroidism: A Danish Population-Based Study. Eur Thyroid J (2014) 3(3):164–72. doi: 10.1159/000365101 PMC4224233 25538898

[12] Page MJ McKenzie JE Bossuyt PM Boutron I Hoffmann TC Mulrow CD . The PRISMA 2020 Statement: An Updated Guideline for Reporting Systematic Reviews. BMJ (2021) 372:n71. doi: 10.1136/bmj.n71 33782057 PMC8005924

[13] Wells GA Shea B O'Connel D Peterson J Welch V Losos M . The Newcastle-Ottawa Scale (NOS) for Assessing the Quailty of Nonrandomized Studies in Meta-Analyses (2009). Available at: http://www.ohrica/programs/clinical_epidemiology/oxford.htm.

[14] Dijkers M . Introducing GRADE: A Systematic Approach to Rating Evidence in Systematic Reviews and to Guideline Development. KT Update (2013) 1(5):1–9.

[15] Yang Y Le Ray I Zhu J Zhang J Hua J Reilly M . Preeclampsia Prevalence, Risk Factors, and Pregnancy Outcomes in Sweden and China. JAMA Netw Open (2021) 3 4(5):e218401. doi: 10.1001/jamanetworkopen.2021.8401 PMC8111481 33970258

[16] Mayrink J Souza RT Feitosa FE Rocha Filho EA Leite DF Vettorazzi J . Incidence and Risk Factors for Preeclampsia in a Cohort of Healthy Nulliparous Pregnant Women: A Nested Case-Control Study. Sci Rep (2019) 2 9(1):9517. doi: 10.1038/s41598-019-46011-3 PMC6606578 31266984

[17] Korevaar TI Steegers EA Chaker L Medici M Jaddoe VW Visser TJ . The Risk of Preeclampsia According to High Thyroid Function in Pregnancy Differs by hCG Concentration. J Clin Endocrinol Metab (2016) 101(12):5037–43. doi: 10.1210/jc.2016-2397 27648965

[18] Chu SY Callaghan WM Kim SY Schmid CH Lau J England LJ . Maternal Obesity and Risk of Gestational Diabetes Mellitus. Diabetes Care (2007) 30(8):2070–6. doi: 10.2337/dc06-2559a 17416786

[19] Miyauchi S Matsuura B Ueda T Eguchi T Tamaru M Yamamoto S . Interleukin-18 Induces Insulin Resistance in the Hyperthyroid State. Endocr J (2013) 60(4):449–55. doi: 10.1507/endocrj.EJ12-0136 23257837

[20] Feodor Nilsson S Andersen PK Strandberg-Larsen K Nybo Andersen AM . Risk Factors for Miscarriage From a Prevention Perspective: A Nationwide Follow-Up Study. BJOG (2014) 121(11):1375–84. doi: 10.1111/1471-0528.12694 24548778

[21] Phoojaroenchanachai M Sriussadaporn S Peerapatdit T Vannasaeng S Nitiyanant W Boonnamsiri V . Effect of Maternal Hyperthyroidism During Late Pregnancy on the Risk of Neonatal Low Birth Weight. Clin Endocrinol (Oxf) (2001) 54(3):365–70. doi: 10.1046/j.1365-2265.2001.01224.x 11298089

[22] Stagnaro-Green A . Overt Hyperthyroidism and Hypothyroidism During Pregnancy. Clin Obstet Gynecol (2011) 54(3):478–87. doi: 10.1097/GRF.0b013e3182272f32 21857178

[23] Downes KL Grantz KL Shenassa ED . Maternal, Labor, Delivery, and Perinatal Outcomes Associated With Placental Abruption: A Systematic Review. Am J Perinatol (2017) 34(10):935–57. doi: 10.1055/s-0037-1599149 PMC5683164 28329897

[24] Boisramé T Sananès N Fritz G Boudier E Aissi G Favre R . Placental Abruption: Risk Factors, Management and Maternal-Fetal Prognosis. Cohort Study Over 10 Years. Eur J Obstet Gynecol Reprod Biol (2014) 179:100–4. doi: 10.1016/j.ejogrb.2014.05.026 24965988

[25] Kassebaum NJ Bertozzi-Villa A Coggeshall MS Shackelford KA Steiner C Heuton KR . Global, Regional, and National Levels and Causes of Maternal Mortality During 1990-2013: A Systematic Analysis for the Global Burden of Disease Study 2013. Lancet (2014) 384(9947):980–1004. doi: 10.1016/S0140-6736(14)60696-6 24797575 PMC4255481

[26] Elbers LPB Fliers E Cannegieter SC . The Influence of Thyroid Function on the Coagulation System and Its Clinical Consequences. J Thromb Haemost (2018) 16(4):634–45. doi: 10.1111/jth.13970 29573126

[27] WHO: Recommended Definitions, Terminology and Format for Statistical Tables Related to the Perinatal Period and Use of a New Certificate for Cause of Perinatal Deaths. Modifications Recommended by FIGO as Amended October 14, 1976. Acta Obstet Gynecol Scand (1977) 56(3):247–53. 560099

[28] Kiserud T Piaggio G Carroli G Widmer M Carvalho J Neerup Jensen L . The World Health Organization Fetal Growth Charts: A Multinational Longitudinal Study of Ultrasound Biometric Measurements and Estimated Fetal Weight. PloS Med (2017) 14(1):e1002220. doi: 10.1371/journal.pmed.1002220 28118360 PMC5261648

[29] Ferrero DM Larson J Jacobsson B Di Renzo GC Norman JE Martin JN Jr . Cross-Country Individual Participant Analysis of 4.1 Million Singleton Births in 5 Countries With Very High Human Development Index Confirms Known Associations But Provides No Biologic Explanation for 2/3 of All Preterm Births. PloS One (2016) 11(9):e0162506. doi: 10.1371/journal.pone.0162506 27622562 PMC5021369

[30] Cellini M Santaguida MG Stramazzo I Capriello S Brusca N Antonelli A . Recurrent Pregnancy Loss in Women With Hashimoto's Thyroiditis With Concurrent Non-Endocrine Autoimmune Disorders. Thyroid (2020) 30(3):457–62. doi: 10.1089/thy.2019.0456 31910128

[31] Casey BM Dashe JS Wells CE McIntire DD Leveno KJ Cunningham FG . Subclinical Hyperthyroidism and Pregnancy Outcomes. Obstet Gynecol (2006) 107(2 Pt 1):337–41. doi: 10.1097/01.AOG.0000197991.64246.9a 16449121

[32] van Dijk MM Smits IH Fliers E Bisschop PH . Maternal Thyrotropin Receptor Antibody Concentration and the Risk of Fetal and Neonatal Thyrotoxicosis: A Systematic Review. Thyroid (2018) 28(2):257–64. doi: 10.1089/thy.2017.0413 29325496

[33] Léger J Delcour C Carel JC . Approach to the Patient Fetal and Neonatal Thyroid Dysfunction. J Clin Endocrinol Metab (2021), 107:dgab747. doi: 10.1210/clinem/dgab747 34636892

[34] Ross DS Burch HB Cooper DS Greenlee MC Laurberg P Maia AL . American Thyroid Association Guidelines for Diagnosis and Management of Hyperthyroidism and Other Causes of Thyrotoxicosis. Thyroid (2016) 26(10):1343–421. doi: 10.1089/thy.2016.0229 27521067

[35] Feki M Omar S Menif O Tanfous NB Slimane H Zouari F . Thyroid Disorders in Pregnancy: Frequency and Association With Selected Diseases and Obstetrical Complications in Tunisian Women. Clin Biochem (2008) 41(12):927–31. doi: 10.1016/j.clinbiochem.2008.05.002 18538668

[36] Männistö T Mendola P Grewal J Xie Y Chen Z Laughon SK . Thyroid Diseases and Adverse Pregnancy Outcomes in a Contemporary US Cohort. J Clin Endocrinol Metab (2013) 98(7):2725–33. doi: 10.1210/jc.2012-4233 PMC3701274 23744409

[37] Medici M Korevaar TI Schalekamp-Timmermans S Gaillard R de Rijke YB Visser WE . Maternal Early-Pregnancy Thyroid Function Is Associated With Subsequent Hypertensive Disorders of Pregnancy: The Generation R Study. J Clin Endocrinol Metab (2014) 99(12):E2591-8. doi: 10.1210/jc.2014-1505 25157540

[38] Moradinazar M Najafi F Nazar ZM Hamzeh B Pasdar Y Shakiba E . Lifetime Prevalence of Abortion and Risk Factors in Women: Evidence From a Cohort Study. J Pregnancy (2020) 2020:4871494. doi: 10.1155/2020/4871494 32395342 PMC7201453

[39] Ohashi M Furukawa S Michikata K Kai K Sameshima H Ikenoue T . Risk-Based Screening for Thyroid Dysfunction During Pregnancy. J Pregnancy (2013) 2013:619718. doi: 10.1155/2013/619718 23606967 PMC3625569

[40] Saki F Dabbaghmanesh MH Ghaemi SZ Forouhari S Ranjbar Omrani G Bakhshayeshkaram M . Thyroid Function in Pregnancy and Its Influences on Maternal and Fetal Outcomes. Int J Endocrinol Metab (2014) 12(4):e19378. doi: 10.5812/ijem.19378 25745488 PMC4338651

[41] Shahid MM Rahman KMT Gomes RR Ferdous M Ferdousi S Zahan T . Association of Gestational Diabetes Mellitus and Thyroid Status During Pregnancy: A Cross-Sectional Study in a Tertiary Health Care Center of Bangladesh. Gynecol Endocrinol (2021) 37(4):312–4. doi: 10.1080/09513590.2020.1866531 33356671

[42] Singh V Natu N Gupta AS . Comparison of Maternal and Perinatal Outcome in Pregnancy With Altered Thyroid Profile and Euthyroid Patients: A Prospective, Observational and Case Control Study in a Tertiary Care Centre. Int J Reprod Contracept Obstet Gynecol (2019) 8(4):1594–600. doi: 10.18203/2320-1770.ijrcog20191224

[43] Su PY Huang K Hao JH Xu YQ Yan SQ Li T . Maternal Thyroid Function in the First Twenty Weeks of Pregnancy and Subsequent Fetal and Infant Development: A Prospective Population-Based Cohort Study in China. J Clin Endocrinol Metab (2011) 96(10):3234–41. doi: 10.1210/jc.2011-0274 21832110

[44] Yang J Liu Y Liu H Zheng H Li X Zhu L . Associations of Maternal Iodine Status and Thyroid Function With Adverse Pregnancy Outcomes in Henan Province of China. J Trace Elem Med Biol (2018) 47:104–10. doi: 10.1016/j.jtemb.2018.01.013 29544795

[45] You SH Cheng PJ Chung TT Kuo CF Wu HM Chu PH . Population-Based Trends and Risk Factors of Early- and Late-Onset Preeclampsia in Taiwan 2001-2014. BMC Pregnancy Childbirth (2018) 18(1):199. doi: 10.1186/s12884-018-1845-7 29855344 PMC5984409

[46] Zhang Y Li Y Shan Z Xu Y Li C Xie X . Association of Overt and Subclinical Hyperthyroidism During Weeks 4-8 With Adverse Pregnancy Outcomes. J Women's Health (Larchmt) (2019) 28(6):842–8. doi: 10.1089/jwh.2018.7180 30855205

